# Supplementary material for: Aircrew rostering workload patterns and associated fatigue and sleepiness scores in short and medium haul flights in Brazil
Source: Sci Rep. 2025 Oct 29;15:37845. doi: 10.1038/s41598-025-21705-z (PMC12572272; doi:10.1038/s41598-025-21705-z)
Supplement: Supplementary file 1 — Supplementary Information. [file 41598_2025_21705_MOESM1_ESM.pdf]

Supplementary Section

1. SAFTE-FAST results for all rosters of 2023 (sample 4)

In this section we present the SAFTE-FAST results for the average values and respective standard errors for the minimum effectiveness EM_C (%), minimum sleep reservoir RM_C (%), and fatigue hazard area FHA_C (min), all computed within critical phases of flight, which include the first and last 30 minutes of each flight sector. The SAFTE-FAST runs were performed with software version 6.6 assuming a 60-minute commuting time from home to station, hotel to station and rest facility to station and vice-versa and the standard SAFTE-FAST Auto-Sleep controls. The details for the SAFTE-FAST parameters and criteria can be found elsewhere[1]. Differently from our previous work that adopted 30-days epochs, the simulations performed here consider a standardized 28-days epoch for each month of 2023, which included duty periods starting after each first day at 0h00 and before the 29th day at 0h00, all in Brasilia time (UTC-3 hours); except for February 2023, where the ending point was chosen as March 1st, 2023 at 0h00 Brasilia Time. The results for all months of 2023 (N = 7149, sample 4) are presented in Table 1.

Period	N	Parameter	EM_C (%)	RM_C (%)	FHA_C (min)
Jan-23	650	Average	73.55	78.17	4.57
		SE	0.20	0.12	0.21
Feb-23	508	Average	75.07	78.95	3.28
		SE	0.23	0.12	0.21
Mar-23	628	Average	75.93	79.11	3.39
		SE	0.27	0.13	0.21
Apr-23	628	Average	76.11	79.22	2.98
		SE	0.26	0.12	0.19
May-23	610	Average	76.43	79.42	2.58
		SE	0.25	0.12	0.19
Jun-23	614	Average	76.18	79.25	2.78
		SE	0.25	0.12	0.19
Jul-23	622	Average	75.04	78.73	4.30
		SE	0.27	0.14	0.28
Aug-23	658	Average	75.97	79.23	2.59
		SE	0.24	0.12	0.16
Sep-23	641	Average	76.20	79.39	2.50
		SE	0.23	0.11	0.16
Oct-23	580	Average	75.69	79.21	3.01
		SE	0.27	0.14	0.19
Nov-23	547	Average	76.17	79.70	2.60
		SE	0.25	0.11	0.16
Dec-23	464	Average	74.95	79.05	3.19
		SE	0.26	0.13	0.19

Table 1. Averages and standard errors (SE) for minimum effectiveness EM_C (%), minimum sleep reservoir RM_C (%) and fatigue hazard area FHA_C (min), calculated within critical phases of flight and 28-days epochs for all rosters of 2023 (sample 4) via the SAFTE-FAST software. Details in the text.

2. Workload factors associated with the highest predicted values of SP and KSS for all rosters of 2023 (sample 4)

Considering all 7149 rosters of 2023, the total duty time, the number of flight sectors and the sum of flight sectors with sit periods longer than one hour were responsible for 25.7(25.6 %), 26.1(20.2 %) and 18.3(14.8 %) of the highest SP(KSS) scores, respectively. The number of night shifts and duties above 9 hours were associated with 15.6(12.3 %) and 10.5(15.3 %) of the predicted peak values of SP(KSS), respectively. The number of sit times longer than one hour and the number of rest periods shorter than 16 hours were associated with 11.9 % and 3.8% of the highest KSS and SP scores, respectively. Table 2 brings the percentages of rosters associated with the workload metrics that produced the highest predicted scores of SP and KSS within 28-days epochs for all rosters of 2023.

In order to estimate the impact of the proposed mitigations for the rostering optimization processes we have calculated the fraction of all 168-hour loops that exceed the thresholds $DT > 44$ h, $N_{CREW} > 15$ and $N_{CREW} + N_{SIT} > 19$. The results are presented in Table 3 for all months of 2023 and show that only a minor fraction of all loops overshoots the proposed mitigations.

Combining the mitigations we found an overall impact of 6.7% considering all 3,610,145 loops of 2023 (7149 rosters).

Period	N	Fatigue/Sleepiness Scale	Workload Metric						
			$DT(h)$	N_{NS}	N_{CREW}	N_{DUTY}	N_{SIT}	N_{REST}	$N_{CREW}+N_{SIT}$
Jan-23	650	SP	25.7	22.0	23.2	8.6	N.A.	3.2	17.2
		KSS	29.5	14.8	18.5	14.2	12.8	N.A.	10.3
Feb-23	508	SP	29.1	13.0	26.0	11.6	N.A.	2.8	17.5
		KSS	26.6	9.8	18.9	17.5	12.0	N.A.	15.2
Mar-23	628	SP	23.9	12.6	24.8	15.8	N.A.	4.3	18.6
		KSS	24.2	13.4	18.0	17.8	11.3	N.A.	15.3
Apr-23	628	SP	24.8	14.2	27.4	9.2	N.A.	4.1	20.2
		KSS	26.4	9.6	20.5	14.5	11.5	N.A.	17.5
May-23	610	SP	25.2	12.3	28.2	10.7	N.A.	4.3	19.3
		KSS	22.6	10.0	22.0	15.6	12.8	N.A.	17.0
Jun-23	614	SP	26.5	15.1	23.9	11.4	N.A.	4.2	18.7
		KSS	25.9	9.6	21.5	15.6	12.2	N.A.	15.1
Jul-23	622	SP	22.2	18.5	28.1	8.5	N.A.	3.4	19.3
		KSS	23.5	14.6	22.5	13.8	10.3	N.A.	15.3
Aug-23	658	SP	24.0	17.0	28.0	7.8	N.A.	4.3	18.9
		KSS	24.8	11.6	21.3	16.4	12.5	N.A.	13.4
Sep-23	641	SP	26.8	13.9	27.3	10.6	N.A.	3.3	18.1
		KSS	24.0	11.9	21.5	15.1	11.7	N.A.	15.8
Oct-23	580	SP	27.2	15.7	26.6	9.8	N.A.	3.3	17.4
		KSS	24.8	14.1	17.6	14.5	13.8	N.A.	15.2
Nov-23	547	SP	28.7	14.4	24.5	11.7	N.A.	4.6	16.1
		KSS	27.1	13.9	17.6	16.5	11.7	N.A.	13.3
Dec-23	464	SP	25.2	18.3	24.1	11.6	N.A.	3.4	17.2
		KSS	28.0	14.0	22.8	11.4	9.5	N.A.	14.2
2023	7149	SP	25.7	15.6	26.1	10.5	N.A.	3.8	18.3
		KSS	25.6	12.3	20.2	15.3	11.9	N.A.	14.8

Table 2. Percentage of rosters (%) associated with the workload metric that produced the highest fatigue (SP) or sleepiness (KSS) estimates within 28-day epochs for all rosters of 2023 (sample 4).

3. Workload metrics within 28-days epochs for all rosters of 2023 (sample 4)

The average values and standard errors of the workload metrics computed within 28-days epochs for all rosters of 2023 are presented in Table 4.

References

1. Rodrigues, T. E. *et al.* Modelling the root causes of fatigue and associated risk factors in the brazilian regular aviation industry. *Saf. Sci.* **157**, 105905 (2023).

Period	N	Mitigating Parameters		
		DT > 44 h	N_{CREW} > 15	$N_{CREW} + N_{SIT}$ > 19
Jan-23	650	4.5	2.4	3.2
Feb-23	508	3.6	2.5	3.6
Mar-23	628	3.7	2.8	4.1
Apr-23	628	5.1	3.5	4.3
May-23	610	4.3	3.7	4.2
Jun-23	614	4.0	3.2	4.5
Jul-23	622	3.6	4.0	4.1
Aug-23	657	3.6	3.1	3.8
Sep-23	641	3.9	3.6	4.2
Oct-23	580	3.7	2.5	3.0
Nov-23	547	3.5	2.7	3.5
Dec-23	464	3.6	2.7	3.1
2023	7149	3.9	3.1	3.8

Table 3. Percentage of all 168-hour loops within each 28-days epochs exceeding the proposed mitigations for DT, N_{CREW} and $N_{CREW} + N_{SIT}$. Also shown the overall percentage of 2023. Details in the text.

Period	N	Parameter	N_{NS}	N_{CNS}	N_{ES}	$DT(h)$	N_{CREW}	N_{WOCL}	N_{SIT}	N_{REST}	N_{DUTY}	$N_{CREW} + N_{SIT}$
Jan-23	650	Average	6.53	2.28	1.75	109.3	31.0	4.50	7.69	5.33	3.93	38.7
		SE	0.10	0.06	0.06	0.8	0.3	0.12	0.14	0.09	0.08	0.5
Feb-23	508	Average	5.50	1.80	1.96	102.2	29.4	3.70	7.87	4.71	3.81	37.3
		SE	0.10	0.06	0.08	1.1	0.4	0.12	0.16	0.10	0.08	0.5
Mar-23	628	Average	5.60	1.74	1.85	102.5	28.9	3.84	7.94	5.06	3.70	36.8
		SE	0.10	0.05	0.06	1.0	0.4	0.12	0.15	0.10	0.08	0.5
Apr-23	628	Average	5.78	1.79	1.71	107.5	30.2	3.86	7.88	5.44	4.00	38.1
		SE	0.10	0.05	0.06	1.0	0.4	0.12	0.15	0.11	0.09	0.5
May-23	610	Average	5.78	1.81	1.68	104.9	30.0	3.64	7.66	5.36	3.78	37.6
		SE	0.10	0.05	0.06	1.0	0.4	0.11	0.14	0.10	0.08	0.5
Jun-23	614	Average	5.97	1.84	1.77	105.3	30.0	3.70	7.91	5.37	3.82	37.9
		SE	0.10	0.05	0.06	1.0	0.4	0.11	0.15	0.11	0.08	0.5
Jul-23	622	Average	6.35	2.04	1.75	106.9	31.8	4.40	7.57	5.44	3.80	39.3
		SE	0.10	0.05	0.06	0.9	0.4	0.13	0.14	0.10	0.08	0.5
Aug-23	658	Average	5.91	1.87	1.63	102.2	29.3	3.77	7.20	5.09	3.74	36.5
		SE	0.10	0.05	0.06	1.0	0.4	0.11	0.14	0.10	0.08	0.5
Sep-23	641	Average	5.81	1.83	1.66	102.5	29.3	3.73	7.29	5.02	3.79	36.6
		SE	0.09	0.05	0.06	1.0	0.4	0.12	0.13	0.10	0.08	0.5
Oct-23	580	Average	5.62	1.77	1.82	103.0	28.7	3.84	7.16	4.94	3.82	35.9
		SE	0.10	0.05	0.07	1.1	0.4	0.12	0.14	0.11	0.08	0.5
Nov-23	547	Average	5.22	1.61	1.85	97.8	27.3	3.37	7.14	4.78	3.35	34.4
		SE	0.11	0.05	0.07	1.1	0.4	0.13	0.15	0.10	0.08	0.5
Dec-23	464	Average	5.75	1.91	2.00	101.0	28.3	4.05	7.10	4.77	3.30	35.4
		SE	0.12	0.06	0.08	1.1	0.5	0.15	0.16	0.11	0.09	0.6

Table 4. Averages and standard errors (SE) of the workload metrics computed within 28-day epochs for all 7149 rosters of 2023 (sample 4).
